# Just-in-Time Encoding Into Visual Working Memory Is Contingent Upon Constant Availability of External Information

**DOI:** 10.5334/joc.364

**Published:** 2024-05-03

**Authors:** Alex J. Hoogerbrugge, Christoph Strauch, Sanne Böing, Tanja C. W. Nijboer, Stefan Van der Stigchel

**Affiliations:** 1 Experimental Psychology, Helmholtz Institute, Utrecht University, Utrecht, The Netherlands

**Keywords:** Visual Working Memory, Copying task, Trade-off, External sampling, Eye movements

## Abstract

Humans maintain an intricate balance between storing information in visual working memory (VWM) and just-in-time sampling of the external world, rooted in a trade-off between the cost of maintaining items in VWM versus retrieving information as it is needed. Previous studies have consistently shown that one prerequisite of just-in-time sampling is a high degree of availability of external information, and that introducing a delay before being able to access information led participants to rely less on the external world and more on VWM. However, these studies manipulated availability in such a manner that the cost of sampling was stable and predictable. It is yet unclear whether participants become less reliant on external information when it is more difficult to factor in the cost of sampling that information. In two experiments, participants copied an example layout from the left to the right side of the screen. In Experiment 1, intermittent occlusion of the example layout led participants to attempt to encode more items per inspection than when the layout was constantly available, but this did not consistently result in more correct placements. However, these findings could potentially be explained by inherent differences in how long the example layout could be viewed. Therefore in Experiment 2, the example layout only became available after a gaze-contingent delay, which could be constant or variable. Here, the introduction of any delay led to increased VWM load compared to no delay, although the degree of variability in the delay did not alter behaviour. These results reaffirm that the nature of when we engage VWM is dynamical, and suggest that any disruption to the continuous availability of external information is the main driver of increased VWM usage relative to whether availability is predictable or not.

## 1 Introduction

Imagine laying a jigsaw puzzle. How many puzzle pieces at a time will you encode into memory for subsequent placement? One might initially expect that working memory is loaded to its capacity every time. However, from personal experience you likely recognize that you rarely apply this strategy, but rather memorize only one or two pieces at a time before trying to place them in their right location.

Representations of visual information from our environment – such as puzzle pieces – are stored in visual working memory (VWM); a short-term, limited-capacity system (Baddeley & Herring, 1983; Ma et al., 2014; Salway & Logie, 1995). The limits of VWM capacity have been studied extensively with change detection paradigms, delayed recall, and various other tasks (e.g., Adam et al., 2017; Brady & Tenenbaum, 2013; Cowan, 2016; Luck & Vogel, 2013; Ma et al., 2014; Oberauer et al., 2018). Typically in such studies, displays with to-be-memorized visual information of certain set sizes (e.g., four coloured squares) are briefly and transiently presented, after which this information does not reappear. After a retention interval, participants are required to report on what they remembered; e.g., they are shown a matching- or non-matching display and report whether any stimuli have changed. VWM capacity is then estimated, for example, from task accuracy on each set size. This line of research has been successful, providing insight into how information is represented in VWM and what its limits are (Cowan, 2016; Luck & Vogel, 2013; Ma et al., 2014).

Interestingly, however, VWM is usually not filled to capacity when participants have the option to look back at the to-be-memorized information. When and how much VWM is loaded as a function of task specifics is therefore part of a growing body of literature (Kristjánsson et al., 2018; O’Regan, 1992; Van der Stigchel, 2020; Wilson, 2002). For example, in a puzzle with four remaining pieces, one might only memorize one or two pieces at a time, fill them in, and then memorize the remaining two pieces in order to fully complete the puzzle. Therefore, VWM can be regarded as being part of a dynamic system that constantly weighs the costs of maintaining a (high) memory load against the costs of external sampling. Indeed, consistent with the example of a jigsaw puzzle, several studies have found participants to minimally utilize VWM in many circumstances where information could be retrieved *just-in-time* from the environment instead (Ballard et al., 1995; Böing et al., 2023; Draschkow et al., 2021; Droll & Hayhoe, 2007; Gajewski & Henderson, 2005; Gray et al., 2006; Hayhoe et al., 2003; Inamdar & Pomplun, 2003; Melnik et al., 2018; Risko & Dunn, 2015; Risko & Gilbert, 2016; Sahakian et al., 2023, 2024; Somai et al., 2020; Triesch et al., 2003). In this just-in-time approach, external information is only fixated and encoded into memory *if* and *when* it is needed for the task at hand, instead of being processed (and memorized) in advance (Droll & Hayhoe, 2007; Hayhoe et al., 2003). Most notably, previous studies either manipulated the distance between the area where external information could be retrieved and the area where that retrieved information needed to be used, or they delayed access to the required external information. When distance was increased, external sampling would theoretically become more costly, since larger (thus more energy-expensive and time-consuming) eye- or head movements were needed to move the gaze back to the external information. Indeed, the cost of sampling altered the trade-off between storing and just-in-time sampling; shorter distances were linked to the dominance of external sampling, whereas larger distances were associated with more storing (Ballard et al., 1995; Draschkow et al., 2021; Inamdar & Pomplun, 2003). The second set of studies delayed the access to external information, for example by letting participants wait every time they wanted to sample externally (Böing et al., 2023; Gray et al., 2006; Melnik et al., 2018; Sahakian et al., 2023; Somai et al., 2020). There, participants showed similar patterns of behaviour as in the distance manipulations, providing strong evidence that the cost of access to information in the external world shifts the balance of *when* and *how much* one relies on VWM.

The aforementioned studies all provided external environments which were predictable or stable: External information was removed after an encoding phase (e.g., change detection tasks) or could be revisited throughout the task (e.g., copying tasks). When external information can be revisited, the just-in-time sampling strategy is especially useful if we can make an estimation of how costly (i.e., time and energy) such a revisit will be. Even if we are aware that we will have to wait two seconds before we can resample information, we can factor that delay into our internal model of the cost of just-in-time sampling versus maintaining higher VWM loads. However, we commonly experience situations in which access to external information is not stable. Think, for example, of intermittent glare from the sun in your eyes while driving, which can make relevant external information (e.g., the position of other cars) unavailable at an unpredictable interval. Or think of loading a web page to look up information; depending on your internet speed it may take a few milliseconds up to several seconds to load the page. In these scenarios, the predictability of access to external information is disrupted. Even though information will sometimes be available instantly, the possibility of a delay may disrupt the ability to factor in the cost of just-in-time sampling. Therefore, one may instead rely on building more elaborate internal representations of the external world whenever it is available, rather than sampling information if and when it is needed. It is yet unclear how disruptions to the predictable availability of external information affect the trade-off between storing and sampling. We here hypothesized that participants minimized VWM usage (i.e., primarily sampled externally) when external visual information was readily available, and that (ir)regularly occluding external information would cause a shift towards internal storage. Furthermore, we asked whether this trade-off would change further as information became less and less predictably available.

Across two experiments, participants performed a copying task in which the required external information was constantly available in one condition, and intermittently occluded to varying degrees in other conditions. In Experiment 1, external information was made available and unavailable for different durations across conditions. Although the pacing was mostly predictable, participants had no control over when external information was made available. Experiment 2 followed up on Experiment 1: External information was unavailable by default and only became available after participants fixated an hourglass for a certain delay period. Importantly, this delay period could be constant (and therefore mostly predictable as in Experiment 1) or variable, which made access to external information less predictable.

## 2 Experiment 1

### 2.1 Methods

#### 2.1.1 Participants and procedure

Sample size was determined based on previous, similar, studies (e.g., Draschkow et al., 2021; Melnik et al., 2018; Somai et al., 2020). 26 participants performed the experiment. Gaze data of one participant was corrupted and two participants stopped early. Of the remaining *23* participants (age range 18–29), 10 indicated female and 13 male gender. All had normal or corrected-to-normal sight. Participants were compensated with €7 per hour or course credits. The experiment was approved by the Faculty Ethics Review Board of the Faculty of Social Sciences, Utrecht University (protocol number 21-0297), adhering to the Declaration of Helsinki.

Participants signed an informed consent form, provided their age category and gender, and were then instructed about the task. Each participant first completed five practice trials in a baseline condition. After confirming that they understood the task, the participant started the actual experiment. The experiment took approximately 90–120 minutes to complete.

#### 2.1.2 Apparatus and stimuli

**Figure 1:**
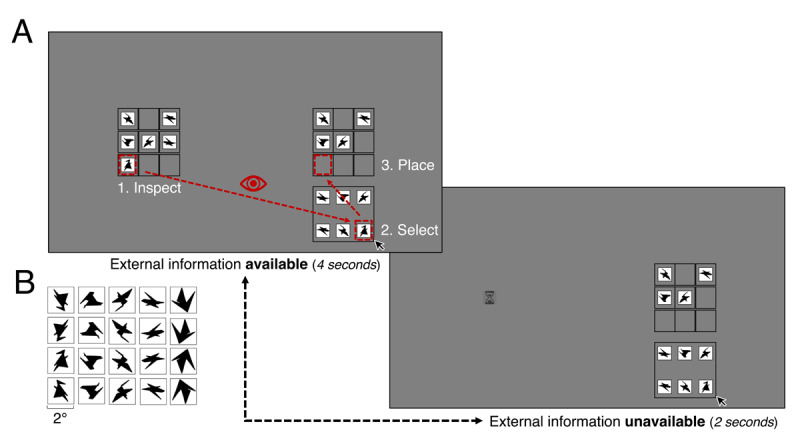
**A.** Example of a partially completed trial in the *High* availability condition in Experiment 1. In this example, four items have already been dragged to their correct position. The example grid alternated between available and unavailable throughout a trial, and this was repeated until the trial was completed. **B.** Stimuli as adapted from Arnoult (1956). In each column there is a unique shape, each mirrored horizontally, vertically, and diagonally; 20 stimuli in total. Each stimulus occupied approximately 2° of visual angle.

Data and code are available on the Open Science Framework https://osf.io/z2n5x/. The experiment was implemented with Python 3.6 and PyGaze (Dalmaijer et al., 2014). The experiment was displayed on a 27 inch LCD monitor (2560 × 1440 pixels, 100 Hz). Participants placed their heads in a fixed chin- and forehead rest at 67.5 centimetres from the screen, such that each 100 × 100 pixel stimulus occupied a visual angle of approximately 2°. The experiment was recorded with an EyeLink 1000 eye tracker (SR Research Ltd., Canada), which measured monocularly at a sampling rate of 1 kHz. We did not standardize whether the left or right eye was tracked. The threshold for eye tracking validation error was 1° (average of 9-point validation) and 1.5° per-point maximum, otherwise the eye tracker was re-calibrated. The stimuli used in this experiment were adapted from Arnoult (1956), and were previously used in Böing et al. (2023), Hoogerbrugge et al. (2023), Sahakian et al. (2023, 2024), and Somai et al. (2020). The stimulus set consisted of five unique shapes, with each shape additionally mirrored horizontally, vertically, and diagonally, creating 20 stimuli in total (Figure 1B).

#### 2.1.3 Task

Participants performed a copying task in which they copied a layout of six stimuli within a 3 × 3 grid on the left side of the screen (*example grid*) to an equally large empty grid on the right side of the screen (*working grid*). The centres of both grids were located at a visual angle of 12° from the centre of the screen, with each of the grids occupying approximately 7.3° × 8.8° of the visual field. In each trial, six stimuli were randomly selected without replacement and the example grid layout was randomly filled with those six stimuli. On the bottom right of the screen, the same stimuli were presented as in the example grid, but in randomized layout (*resource grid*; Figure 1A).

The participants’ task was to exactly recreate the layout of the example grid in the working grid, by dragging stimuli from the resource grid to their correct location in the working grid (Figure 1A). After correctly placing a stimulus, that working grid location would briefly flash green (750 ms). After incorrectly placing a stimulus, the corresponding grid location would flash red (750 ms) and the stimulus would not lock into place, but instead fly back to the location from which it was dragged. The stimuli in the resource grid remained visible for the whole duration of the trial, even when already placed correctly in the working grid. A trial ended whenever the grid was fully copied or if the task was not completed after 42 seconds. Participants were shown feedback (“Correct”/”Timed out”) after each trial.

In the *baseline* condition, the example grid was always visible. In the other three conditions, the example grid was either visible or occluded at specified intervals throughout a trial. Namely, the example grid was repetitiously (1) visible for 4 seconds and subsequently occluded for 2 seconds, such that the availability of external information was *High*; (2) visible for 3 seconds and occluded for 3 seconds, such that the availability of external information was *Medium*; (3) visible for 2 seconds and occluded for 4 seconds, such that the availability of external information was *Low*.

In each trial, the occlusion time was multiplied by a noise factor drawn from a Gaussian distribution (*μ* = 1.0, *σ* = .1), with the visible time being adjusted accordingly such that the sum of visible time and occlusion time was always 6 seconds. Because the occlusion time was multiplied by a Gaussian noise factor, the possible variation of occlusion times was greater in the *Low* availability condition than in the *High* availability condition – thereby making occlusion durations somewhat less predictable. Whenever the example grid was occluded, a pictogram of an hourglass would appear in its place. The example grid was visible at the start of each trial. Visibility and occlusion of the example grid were repeated until the trial ended. Example videos of trials can be found on OSF (https://osf.io/kz5v6/).

Each of the four conditions was tested in its own block of 35 trials and the block order was randomized between participants. The eye tracker was calibrated and validated before the start of each block. Additionally, a drift check was performed before the start of each trial, by computing the root mean squared error (RMS) between the gaze prediction and a central fixation cross which was shown for two seconds. If the RMS was greater than 1.5° for more than two subsequent trials, the experimenter would recalibrate.

#### 2.1.4 Outcome variables

We computed outcome variables to provide an estimate of how much information was encoded into VWM, and subsequently placed, for each inspection of the example grid. **(A)** The number of *example grid inspections*, which was calculated by counting how many times within a trial the participant made a saccade across the centre of the screen from the right side to the left side. In effect, this variable represents how often participants sampled externally by looking toward the example grid after focusing on the working- and resource area. We did not count crossings in which only the hourglass was fixated, and assumed that short fixations would be unlikely to allow for meaningful encoding (e.g., Bays et al., 2011). Therefore an inspection would only be counted if the example grid was viewed for at least 120 ms before the participant crossed back towards the working- and resource area. **(B)** The number of *fixations per inspection* was computed by dividing the number of fixations within the boundaries of the example grid by the number of useful inspections. This variable approximates how much information participants attempted to take in each time they placed their overt attention on the example grid. **(C)** The number of correct *items placed per inspection* was computed by dividing the number of correctly placed items per trial by the number of useful inspections made in that trial. It is an estimate of how many items participants (accurately) encoded during each inspection.

We report three additional outcome variables: **(D)** *Completion time (seconds)* was calculated from the start of the trial until all items were placed correctly, or until the 42-second timer was reached. Because the periods during which the example grid was occluded were not useless to participants (i.e., they could still place items during that time), only the time spent gazing at the hourglass in the location of the occluded example grid was subtracted from the completion time. **(E)** The number of *errors per trial*, in which an error constituted the attempted placement of any item in an incorrect slot in the working grid. A greater number of errors may reflect that items were encoded less accurately (Koevoet et al., 2023; van den Berg et al., 2012) or that participants had more liberal thresholds for the quality of memory representations that they were willing to act on (Sahakian et al., 2023). **(F)** The *proportion spent waiting* was expressed as the duration that participants spent gazing at the hourglass, divided by the actual duration with which the example grid was occluded during that trial. This measure effectively reflects the proportion of a trial that participants spent unproductively waiting. For example: In a trial in the Low condition, if the example grid was occluded for 12 seconds in total and a participant spent 600 ms gazing at the hourglass, the proportion spent waiting is 0.05. In the High condition, if the grid was occluded for 6 seconds in total and a participant spent 300 ms gazing at the hourglass, the proportion spent waiting is also 0.05. As such, the proportion that participants spent waiting was standardized between 0 and 1 and could be compared between conditions.

#### 2.1.5 Analyses

Fixations were detected using I2MC (Hessels et al., 2017). All fixation candidates shorter than 60 ms were removed, and fixation candidates which were separated by less than 1° distance were merged. This approach has been shown to remove variation between fixation detection algorithms (Hooge et al., 2022).

Statistical analyses were conducted with JASP 0.18.3 (JASP Team, 2022). The six outcome variables were aggregated per participant, per condition. All were aggregated by the mean, except for *completion time*, which was aggregated by the median. In order to test whether availability of external information affected our outcome variables, we report Repeated Measures ANOVAs. If the assumption of sphericity was violated for an outcome variable, we report corrected ANOVAs (Greenhouse-Geisser if *ϵ* < .75, otherwise Huynh-Feldt; following Abdi, 2010). Effect sizes of ANOVAs are reported with *η*^2^. Post-hoc, paired samples t-tests are reported, and *p*-values were Bonferroni corrected for six comparisons within each variable. Effect sizes of t-tests are reported with Cohen’s *d*. All statistical test outcomes including Bayes Factors are reported on the Open Science Framework.

### 2.2 Results

#### 2.2.1 Example grid inspections, fixations, and items placed per inspection

**Figure 2:**
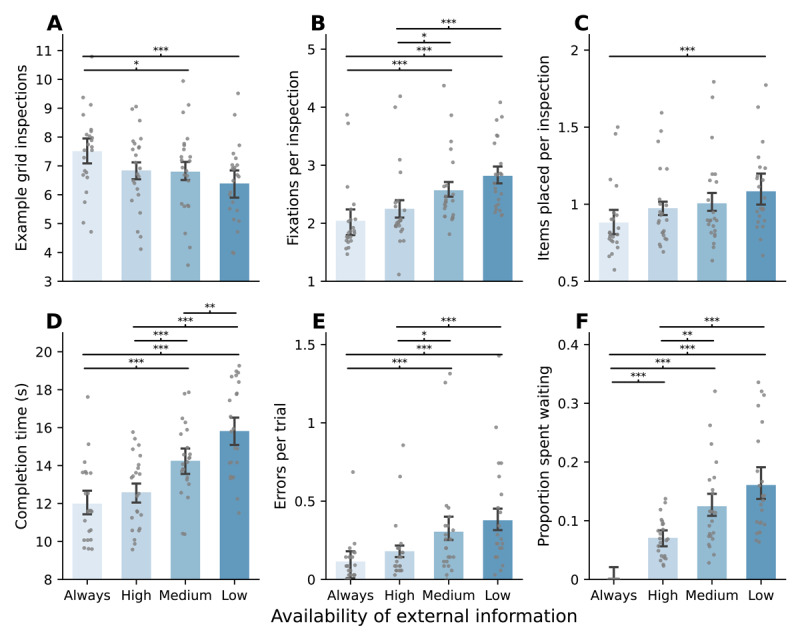
Barplots (mean ± 95% within-subjects CI) for each variable, per condition. Individual points represent within-participant aggregates. **A.** The average number of inspections of the example grid per trial. **B.** The average number of fixations made on the example grid per inspection. **C.** The average number of correctly placed items per inspection. **D.** The median completion time (in seconds). Time spent fixating at the example grid while it was occluded was subtracted. **E.** The average number of incorrectly placed items per trial. **F.** The average proportion spent fixating at the example grid location while it was occluded. *Note*: Post-hoc paired samples t-tests (Bonferroni corrected); *p < .05, **p < .01, ***p < .001.

In the baseline condition, participants made a median of 7.66 (*median absolute deviation; MAD* = 1.03) example grid inspections per trial, meaning they sampled externally more than once per item (Figure 2A). They inspected the example grid less often when availability of visual information was lower, *F*(2.7, 59.5) = 6.97, *p* < .001, *η*^2^ = 0.24. This main effect was primarily driven by the difference between the baseline and the decreased access conditions, which indicates that the disruption to constant availability was the main driver of increased memory usage. When availability of external information was further reduced, no effect on the number of inspections was found; even in the Low availability condition, participants still inspected the example grid a median of 6.63 (*MAD* = 1.06) times – a small difference compared to the High availability condition (*Mdn* = 6.91, *MAD* = 1.07).

Although the number of example grid inspections was mainly different between the baseline and the decreased access conditions, the number of fixations per example grid inspection steadily increased as availability was reduced (*F*(2.1, 46.4) = 21.91, *p* < .001, *η*^2^ = 0.5). This indicates that participants at least *attempted* to encode more information per inspection, ranging between *Mdn* = 1.83(*MAD* = 0.40) fixations in the baseline condition, up to *Mdn* = 2.69(*MAD* = 0.48) fixations in the Low condition (Figure 2B).

This increase in the number of fixations was somewhat reflected in the number of correct placements per inspection (Figure 2C). There was an overall increase across conditions (*F*(2.0, 43.8) = 6.60, *p* = .003, *η*^2^ = 0.23), although the number of items placed was relatively low overall and post-hoc tests only showed a significant difference (*p* < .001) between the baseline condition and the lowest-availability condition. In the baseline condition, participants correctly placed just less than one item per inspection (*Mdn* = 0.80, *MAD* = 0.16), and interestingly this stayed below one item even in the Low availability condition (*Mdn* = 0.97, *MAD* = 0.21).

The number of example grid inspections and the number of fixations and correct placements per inspection tell us that participants encoded and subsequently placed (slightly less than) one item per crossing, when external information was always available. Removing the ability to always inspect was the main driver of fewer inspections of external information, and an increased number of placements per inspection. When the example grid availability was further decreased, participants did not inspect it less frequently, but they inspected it with more fixations. While participants changed their eye movement strategy, this did not clearly translate into a different number of correct placements per inspection: participants seemed to attempt to *encode* more, but they did not necessarily *place* more items afterwards or make fewer errors. Furthermore, participants still did not regularly place two (or more) items after inspection, even when the example grid was effectively occluded for two-thirds of a trial.

#### 2.2.2 Completion time, errors, and proportion spent waiting

All participants were able to consistently complete the task within the 42-second time limit. However, they seemed not to (be able to) alter their strategy enough to keep completion times consistent across the decreased availability conditions. Participants took longer to finish the task across almost all conditions as availability decreased (*F*(3, 66) = 35.97, *p* < .001, *η*^2^ = 0.62), except between the baseline and the High availability condition (*p* = 0.88; Figure 2D). Participants made more incorrect placements as availability of external information was reduced (*F*(1.8, 39.7) = 15.62, *p* < .001, *η*^2^ = 0.42), ranging from a median of 0.09(*MAD* = 0.09) errors per trial in the baseline condition to 0.23 (*MAD* = 0.26) errors per trial in the Low reliability condition (Figure 2E).

Interestingly, participants spent an increasing proportion of trials doing nothing, as the example grid was occluded for longer periods, *F*(1.7, 37.0) = 52.69, *p* < .001, *η*^2^ = 0.71 (Figure 2F). In the Low availability condition, participants spent a median proportion of 0.13 (*MAD* = 0.07) gazing at the hourglass in the location of the occluded information – amounting to around one second of waiting as occlusion lasted 10.17 seconds on median in the Low condition. These findings suggest that, although participants attempted to memorize and place more items as availability was decreased, this adaptation was not necessarily time-efficient; they did not compensate for the decreased availability enough to avoid waiting unproductively.

### 2.3 Interim discussion

We here set out to disrupt the ability to just-in-time sample external information by intermittently occluding the example grid. When the visual information required to perform the task was always available, participants memorized and placed just under one item per inspection of external information, consistent with findings from similar paradigms (Sahakian et al., 2023; Somai et al., 2020). When external information was not continuously available throughout a trial, participants adapted their strategy and inspected the example grid less often but with more fixations, which implies that they at least attempted to increase VWM usage. Interestingly, however, the number of placed items did not strongly increase when external availability was decreased: participants placed approximately the same number of items, irrespective of the degree of availability. This provides evidence that the trade-off can be influenced by disrupting participants’ ability to sample external information just-in-time, and that it is nonlinear in nature: *Any* removal of self-pacing shifts the trade-off, and this removal seems to influence it more heavily than further decreases in availability of external information.

However, in the current manipulation participants had limited time to view the example grid, which possibly influenced the amount of external information that could be encoded per inspection (see General Discussion; Koevoet et al., 2023). Furthermore, Experiment 1 did not specifically test the removal of self-pacing while keeping other parameters intact. For instance, the increased occlusion duration indirectly introduced a delay of availability, which has been shown to influence the trade-off (Böing et al., 2023; Gray et al., 2006; Melnik et al., 2018; Sahakian et al., 2023; Somai et al., 2020) and may have thus confounded the manipulation. Additionally, the example grid was incidentally available at the right time, regardless of condition, as evidenced by the generally small proportions of trials spent waiting. This means that the just-in-time aspect of availability was partially left intact.

In Experiment 2 we therefore manipulated availability of the example grid without altering the average occlusion durations across conditions. Furthermore, we did not limit how long participants could view the example grid. As in Experiment 1, we expected that participants would predominantly rely on external sampling when external information was readily available. By introducing a delay we expected to observe a shift in the trade-off towards stronger reliance on internal storage in VWM. Critically, we expected that adding variability to the delay period would cause participants to rely even less on external sampling and to encode more items per example grid inspection.

## 3 Experiment 2

### 3.1 Methods

The methods in Experiment 2 were the same as in Experiment 1, unless stated differently.

#### 3.1.1 Participants

16 participants performed the experiment; none of whom had participated in Experiment 1. Gaze data of one participant was corrupted. Of the remaining 15 participants (age range 19–44; *M* = 25.2), 11 indicated female and 4 indicated male gender.

#### 3.1.2 Task

In Experiment 2, the example grid was occluded by default and showed only if participants gazed at the example grid area for a certain amount of time. Again, the delay was signaled by an hourglass. When the delay period was served, the example grid remained available for as long as participants gazed at it.

In the *baseline* condition, the example grid showed without delay after participants’ gaze was detected in that area. In the *constant delay* condition, participants had to gaze at the hourglass for exactly two seconds before the example grid appeared. In the *low variability* condition, the delay period could range between 0 and 4 seconds, drawn from a Gaussian distribution (*μ* = 2.0s, *σ* = 0.1s); in the *high variability* condition, the delay period could also range between 0 and 4 seconds, but was drawn from a wider Gaussian distribution (*μ* = 2.0s, *σ* = 1.0s). In the variable delay conditions, a new delay duration would be drawn after each time the delay period was fully served – meaning that the delay duration changed multiple times within a trial (see Supplementary Material Figure 1 for the generated distributions of delay durations). Importantly, on average the delay period was similar across the three non-baseline conditions, thereby ensuring that any behavioural differences between conditions would be caused by uncertainty regarding availability, and not by the inherent difference in delay duration.

Each of the four conditions was tested in its own block of 35 trials and block order was randomized. Participants were instructed before the start of each block whether there was “immediate availability”, “a constant delay”, “some variance”, or “a lot of variance”.

#### 3.1.3 Analyses

Instead of the proportion spent waiting, we report **(F)** The *time spent waiting* in seconds. The time spent waiting represents how long participants gazed at the hourglass while the example grid was occluded, and provides an indication whether overall delay durations were similar between conditions in which a delay was present. This outcome variable was aggregated by the median per participant, per condition.

### 3.2 Results

#### 3.2.1 Example grid inspections, fixations, and items placed per inspection

**Figure 3:**
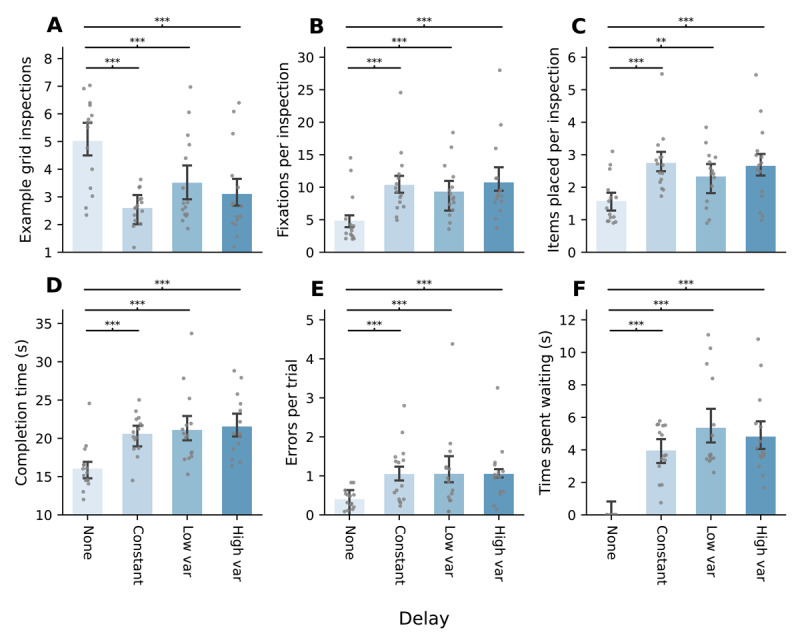
Barplots (mean ± 95% within-subjects CI) for each variable, per condition. Individual points represent within-participant aggregates. **A.** The average number of inspections of the example grid per trial. **B.** The average number of fixations made on the example grid per inspection. **C.** The average number of correctly placed items per inspection. **D.** The median completion time (in seconds). Time spent fixating at the example grid while it was occluded was subtracted. **E.** The average number of incorrectly placed items per trial. **F.** The median time per trial spent fixating at the example grid location while serving the delay period. *Note*: Post-hoc paired samples t-tests (Bonferroni corrected); *p < .05, **p < .01, ***p < .001.

Participants inspected the example grid a median of 5.69 (*MAD* = 1.34) times per trial when there was no delay. Although there was an overall effect of condition on the number of inspections (*F*(3, 42) = 16.92, *p* < .001, *η*^2^ = 0.55), introducing any delay was the main driver of significantly decreased inspections (all *p* < .001 compared to no delay), but whether the delay was constant or variable did not further affect the number of inspections significantly (Figure 3A).

Similar results were observed for the number of fixations per inspection (*F*(1.8, 24.5) = 14.27, *p* < .001, *η*^2^ = 0.51); participants likely attempted to encode more items when there was a delay compared to no delay (all *p* < .001), but again there was no effect of whether the delay period could vary (Figure 3B).

The effect of introducing a delay was also reflected in the number of items placed per inspection (*F*(3, 42) = 11.77, *p* < .001, *η*^2^ = 0.46). Participants placed slightly more than one item (*Mdn* = 1.35, *MAD* = 0.56) per inspection when appearance of the example grid was not delayed, and placed *Mdn* = 2.41 to *Mdn* = 2.68 items when a delay was introduced (all *p* < .01). However, the number of placements did not differ significantly between any of the delay conditions (Figure 3C).

#### 3.2.2 Completion time, errors, and time spent waiting

All participants could consistently complete trials within the 42-second time limit. Participants took *Mdn* = 15.53 (*MAD* = 2.01) seconds to complete the task when there was no delay. When a delay was introduced, median completion time increased to 20.82, 20.37 and 20.58 seconds for the constant delay, low variance, and high variance conditions respectively (all *p* < .001 compared to no delay). Despite an overall effect (*F*(3, 42) = 16.45, *p* < .001, *η*^2^ = 0.54), there were no significant differences between delay conditions (Figure 3D).

Participants made less than one error per trial when there was no delay (*Mdn* = 0.31, *MAD* = 0.20), and this differed significantly from the delay conditions in which they made nearer to one error per trial (*Mdn* = 0.74, 0.86 and 1.00, respectively; all *p* < .001). Again, there was an overall effect (*F*(1.6, 22.4) = 8.46, *p* = .003, *η*^2^ = 0.38), but no further difference in the number of errors per trial between the delay conditions (Figure 3E).

Across the three delay conditions, participants spent an equal amount of time per trial waiting for the example grid to reappear, *F*(2, 28) = 3.12, *p* = .060, *η*^2^ = 0.18 (*Mdn* = 3.68, 3.68 and 3.85 seconds, respectively; Figure 3F). This indicates that there were no inherent differences in delay duration across those three conditions.

#### 3.2.3 Inspection- and build time

Because the delay itself was excluded from the overall completion time, the current findings show that participants were generally faster at completing the task than when there was no delay at all. In order to investigate the cause of this increase, we split completion time into its two constituent parts; **(A)** Total inspection time in seconds, computed as the sum fixation duration on the example grid (excluding waiting time); **(B)** Total build time in seconds, computed as the sum fixation duration on the right-hand side of the screen.

**Figure 4:**
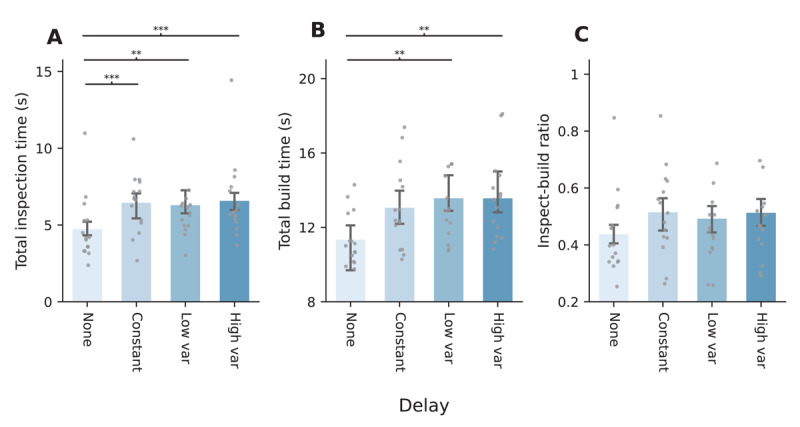
Barplots (mean ± 95% within-subjects CI) for each variable, per condition. Individual points represent within-participant aggregates. **A.** The median time spent inspecting the example grid, in seconds. **B.** The median time spent building on the right side of the screen, in seconds. **C.** The ratio between time spent inspecting and time spent building. *Note*: Post-hoc paired samples t-tests (Bonferroni corrected); *p < .05, **p < .01, ***p < .001.

In the delay conditions, participants spent an increased amount of time inspecting the example grid compared to the conditions without delay (all *p* < .01), but spent equally long across the delay conditions (*F*(2, 28) = 0.20, *p* = .819, *η*^2^ = 0.01; Figure 4A).

Similarly, participants spent more time building the grid in the low- and high-variance conditions than in the condition without delay (*p* = .007 for both, respectively; Figure 4B). As such, increased completion times were caused by increased inspection times as well as increased build times.

Higher memory loads (from increased inspection durations) thus went paired with increased build times, and the proportion of inspection time relative to build time did not change much across conditions; participants spent approximately half as much time encoding information as they spent using that information. Although the ratio between inspecting and building was significantly different between conditions in general (*F*(3, 42) = 2.93, *p* = .045, *η*^2^ = 0.17), none of the post-hoc t-tests showed significant differences (Figure 4C).

## 4 General discussion

It has been established that, under full and constant availability of external information, participants predominantly reduce VWM load and sample information from the environment only if and when they need that information for the task at hand. When the cost of sampling from the environment is increased (in previous studies this took the form of a delay or increased distance), participants’ strategy shifts towards storing relatively more items in memory compared to when the cost of sampling is low. However, in previous studies the cost of sampling was generally stable and predictable, meaning that the cost of sampling could be factored in to the trade-off between just-in-time external sampling and internal storing in VWM. Here, we investigated whether external sampling remains the dominant strategy when the ability to make accurate estimations of the availability of external information is reduced. To test this, we let participants perform a copying task in which external information became available independent of participants’ interaction with it (Experiment 1) and in which access to external information was less predictable (Experiment 2).

In Experiment 1, we intermittently showed and occluded the required external information and participants had no control over this pacing. When the external information was always available, participants memorized and placed just under one item per inspection of external information, indicating a preference to rely on the external world for just-in-time sampling per default, consistent with earlier findings (e.g., Sahakian et al., 2023; Somai et al., 2020). When external information was made less frequently available, participants attempted to encode more information into VWM per inspection of the example grid, although this did not necessarily reflect in more items placed. Notably, participants performed worse as external information was occluded for greater proportions of trials; not only did they take longer to complete the task, they also made more errors and spent more time waiting unproductively.

However, it was unclear whether the observed behaviour was the result of a reluctance to encode more information, or whether participants did not have enough time to do so. In Experiment 2 we therefore introduced a gaze-contingent delay before external information was made available. This delay could be consistently two seconds, or be drawn from a narrow or wide Gaussian distribution centered around two seconds. Participants relied heavily on external sampling when the required information was easily accessible (no delay), and shifted towards using more internal storage when a two-second delay was introduced (conceptually reproducing e.g., Böing et al., 2023; Sahakian et al., 2023; Somai et al., 2020). The trade-off did not shift further with the introduction of variability in the delay. Given that the delay in the high-variance condition ranged between 0 and 4 seconds (see Supplementary Material Figure 1), it is unlikely that the variability of the delay was insufficiently efficacious to reveal meaningful variability-caused effects. Rather, we consider it most likely that predictability of availability does not influence the trade-off between internal storage and external sampling. Additional Bayesian statistics provide moderate evidence for no modulation between the delay conditions on our outcome measures (see Supplementary Material Tables 2 & 3).

Notably, participants relied more on memory in Experiment 2 than in Experiment 1. Due to the forced delay in Experiment 2, participants may have experienced greater time pressure than in Experiment 1, leading them to encode more information whenever it was available. However, only 53 out of 2,097 trials (2.5%) exceeded the time limit in Experiment 2, which makes it unlikely that time pressure was perceived as very high. More likely, this difference in reliance on memory may be explained by the fact that participants had limited time to view the example grid in Experiment 1, whereas they could inspect the example grid for as long as they wanted in Experiment 2. As a result, participants could encode (and subsequently place) more stimuli per inspection than they could in Experiment 1. This is particularly reflected in the number of fixations per inspection: Participants often made more than six fixations, even though there were only six stimuli to encode. This means that they fixated some stimuli multiple times, indicative of more elaborate encoding (e.g., rehearsing or reinstating; Alfandari et al., 2019; Meghanathan et al., 2019; Zelinsky et al., 2011).

The low number of items placed in Experiment 1 could also be due to the stimuli being complex, making them relatively difficult to encode and maintain in VWM (Bethell-Fox & Shepard, 1988; Eng et al., 2005), especially given that viewing time was limited and somewhat unpredictable (Bays et al., 2011; de Jong et al., 2023). The current stimulus set in combination with limited viewing time may have caused ceiling-effects of participants’ ability to encode items. Using simpler stimuli may have led to decreased sampling behaviour and more items placed per inspection (cf. Hoogerbrugge et al., 2023). Avoiding ceiling effects could provide a more sensitive measure of the effect of availability of external information on memory strategies – not only in terms of the number of items memorized, but also on the origin of incorrect placements (Oberauer et al., 2018).

In Experiment 1, participants had no control over availability of the example grid and could not self-initiate the occlusion period, which could have contributed to the relatively low memory usage in Experiment 1 compared to Experiment 2. Namely, participants may have been hampered in their ability to prepare for a shift of attention in the periods just before external information became available (reminiscent of task switching costs; Nieuwenhuis & Monsell, 2002; Rogers & Monsell, 1995; Rubinstein et al., 2001). This lack of preparedness may have affected how much information participants *could* encode. Previous work showed that tonic alerting is linked to how much participants (can) encode on a copying task (Koevoet et al., 2023); participants placed more items correctly when their state of alertness was higher before encoding than when it was low. This idea fits with the finding that participants encoded more items in Experiment 2 (in which they could prepare to encode), and strengthens our theory that participants prefer to access external information when they need it and are ready to process it. When exactly these states occur, how the brain monitors for this readiness, and how this depends on one’s active interaction with external information will require further investigation.

Additionally, we found several inspections of external information per trial without subsequent placement of any items (note that these were *useful* inspections, during which external information was at least briefly viewed). Qualitatively, these inspections occurred somewhat more frequently at the start of trials, but were otherwise evenly distributed throughout trials. Although the current paradigm might not be sufficiently sensitive to attain a complete understanding of why these crossings were made, we speculate that they were explorative or comparative in nature; participants briefly inspected the whole environment before starting copying (forming an initial strategy), or later briefly checked which items they had not placed yet. We suggest that future research further investigates why these inspections occur, taking into account the theories that differing aspects of external information may be gathered during inspections (e.g., features, locations, and/or chunks; Ballard et al., 1995; Huang & Awh, 2018), and that memory may not always be completely depleted before a new inspection is made (e.g., Ballard et al., 1995; Sahakian et al., 2023).

Furthermore, completion times were longer in the non-baseline conditions in both experiments, even though actual waiting time was subtracted from this measure. What led to this temporal inefficiency beyond waiting alone? In the delay conditions of Experiment 2, participants spent more time inspecting the example grid as well as more time building the layout than in the baseline condition. Upon closer inspection, the observed completion times in Experiment 2 were positively linked to the number of inspections, the number of fixations per inspection, as well as the number of errors per trial, all of which cost time (see Supplementary Material Table 4). Participants also made longer fixations, and fewer fixations per second in delay conditions (Supplementary Material Figure 2), which indicates more time spent encoding (Bays et al., 2011; Hoogerbrugge et al., 2023), and has been linked to higher VWM load as well as general cognitive load (Meghanathan et al., 2015; Mills et al., 2011; Woodman et al., 2001). These findings indicate that the external availability of information benefits the speed with which we can execute tasks (cf. Hoogerbrugge et al., 2023) relative to when availability is delayed, even when correcting for delay durations.

In natural settings, we rarely fully load VWM to capacity (Van der Stigchel, 2020), and as such the current paradigm is not directly aimed at, nor suited for, making statements about the capacity limits of VWM (Oberauer et al., 2018). Rather, the current paradigm allows one to investigate how VWM is used in more naturalistic and noisy settings, where strategies, preferences, and executive functioning, amongst others, all play essential roles. During the task, participants are required to encode content into VWM, perform a search task in the pool of available stimuli, and perform actions with the items that they find – all while maintaining a mental map of which items have been placed and which have not. As such, working memory must be utilized in multiple formats (i.e., visual representations, spatial locations, etc.) and content must be utilized in multiple modalities (i.e., item recognition, recall, memory updating; Oberauer et al., 2018). When provided with such complexity, introducing any additional working memory load may introduce undesired noise (e.g., Bays, 2014; Oberauer & Lin, 2017; Schurgin et al., 2020), thereby making the task not only more effortful, but also more prone to mistakes. We here focused on manipulating the ability to sample just-in-time, but it is clear that the paradigm provides a rich environment in which to study different aspects of working memory and to place them within a formalized model (Ngiam, 2023).

In sum, we here investigated how the trade-off between storing in visual working memory versus sampling from the external world shifts, as the ability to sample external information just-in-time was manipulated. Generally, any disruption to the continuous availability of external information, such as intermittent occlusion or a delay period, were the main drivers of increased memory usage. There was no consistent evidence that further manipulations of the frequency or predictability with which information became available affected the storage-sampling trade-off. These findings suggest that the cost of external sampling is primarily driven by time costs rather than predictability of those time costs.

## Data Accessibility Statement

All code and data can be retrieved from the Open Science Framework https://osf.io/z2n5x/.

## Additional File

The additional file for this article can be found as follows:

10.5334/joc.364.s1 Supplementary Material. Supplementary Figure 1: Distribution of generated delay durations in Experiment 2. Supplementary Table 1: Outcome measures used for analysis of both experiments. Supplementary Table 2: Outcomes of Bayesian Repeated-Measures ANOVAs in Experiment 2. Supplementary Table 3: Statistical outcomes for Bayesian paired samples t-tests in Experiment 2. Supplementary Figure 2: Barplots of fixations per second and fixation duration in Experiment 2. Supplementary Table 4: Outcomes of Linear Mixed Effect model.
