# Supplementary material for: Just-in-Time Encoding Into Visual Working Memory Is Contingent Upon Constant Availability of External Information
Source: J Cogn. 2024 May 3;7(1):39. doi: 10.5334/joc.364 (PMC11067970; doi:10.5334/joc.364)
Supplement: Supplementary Material. — Supplementary Figure 1: Distribution of generated delay durations in Experiment 2. Supplementary Table 1: Outcome measures used for analysis of both experiments. Supplementary Table 2: Outcomes of Bayesian Repeated-Measures ANOVAs in Experiment 2. Supplementary Table 3: Statistical outcomes for Bayesian paired samples t-tests in Experiment 2. Supplementary Figure 2: Barplots of fixations per second and fixation duration in Experiment 2. Supplementary Table 4: Outcomes of Linear Mixed Effect model. [file joc-7-1-364-s1.pdf]

SUPPLEMENTARY MATERIAL TO: JUST-IN-TIME ENCODING INTO VISUAL WORKING MEMORY IS CONTINGENT UPON CONSTANT AVAILABILITY OF EXTERNAL INFORMATION

Alex J. Hoogerbrugge[*]
Experimental Psychology
Helmholtz Institute, Utrecht University
Utrecht, The Netherlands
a.j.hoogerbrugge@uu.nl

Christoph Strauch
Experimental Psychology
Helmholtz Institute, Utrecht University
Utrecht, The Netherlands

Sanne Böing
Experimental Psychology
Helmholtz Institute, Utrecht University
Utrecht, The Netherlands

Tanja C. W. Nijboer
Experimental Psychology
Helmholtz Institute, Utrecht University
Utrecht, The Netherlands

Stefan Van der Stigchel
Experimental Psychology
Helmholtz Institute, Utrecht University
Utrecht, The Netherlands

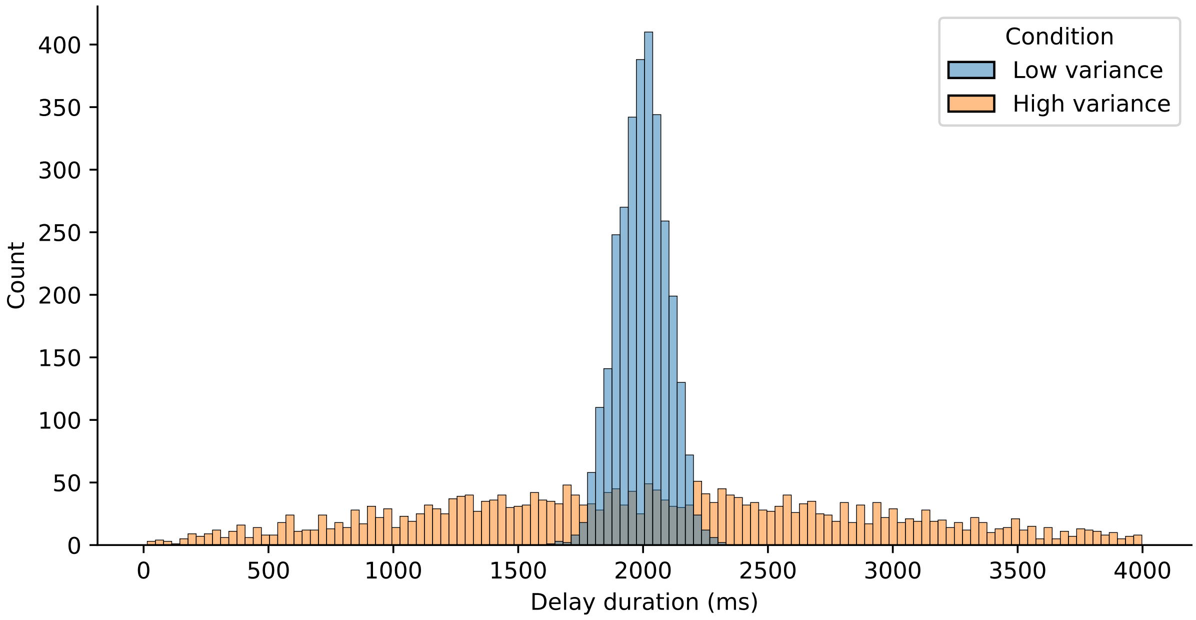

Figure 1: Distribution of generated delay durations in Experiment 2.

[*]Corresponding author

Table 1: Outcome measures used for analysis of both experiments.

	Outcome variable	Description
**A**	Example grid inspections	Calculated by counting how many times within a trial the participant made a saccade across the centre of the screen from the right side to the left side. In effect, this variable represents how often participants sampled externally by looking toward the example grid after focusing on the working- and resource area. We did not count crossings in which only the hourglass was fixated, and assumed that short fixations would be unlikely to allow for meaningful encoding (e.g., Bays et al., 2011). Therefore an inspection would only be counted if the example grid was viewed for at least 120ms before the participant crossed back towards the working- and resource area.
**B**	Fixations per inspection	Computed by dividing the number of fixations within the boundaries of the example grid by the number of useful inspections. This variable approximates how much information participants attempted to take in each time they placed their overt attention on the example grid.
**C**	Items placed per inspection	Computed by dividing the number of correctly placed items per trial by the number of useful inspections made in that trial. It is an estimate of how many items participants (accurately) encoded during each inspection.
**D**	Completion time (s)	Calculated from the start of the trial until all items were placed correctly, or until the 42-second timer was reached. Because the periods during which the example grid was occluded were not useless to participants (i.e., they could still place items during that time), only the time spent gazing at the hourglass in the location of the occluded example grid was subtracted from the completion time.
**E**	Errors per trial	An error constituted the attempted placement of any item in an incorrect slot in the working grid. A greater number of errors may reflect that items were encoded less accurately (Koevoet et al., 2023; van den Berg et al., 2012) or that participants had more liberal thresholds for the quality of memory representations that they were willing to act on (Sahakian et al., 2023).
$\mathbf{F}_{Exp1}$	Proportion spent waiting	Expressed as the duration that participants spent gazing at the hourglass, divided by the actual duration with which the example grid was occluded during that trial. This measure effectively reflects the proportion of a trial that participants spent unproductively waiting. For example: In a trial in the Low condition, if the example grid was occluded for 12 seconds in total and a participant spent 600 ms gazing at the hourglass, the proportion spent waiting is 0.05. In the High condition, if the grid was occluded for 6 seconds in total and a participant spent 300 ms gazing at the hourglass, the proportion spent waiting is also 0.05. As such, the proportion that participants spent waiting was standardized between 0 and 1 and could be compared between conditions.
$\mathbf{F}_{Exp2}$	Time spent waiting (s)	Represents how long participants gazed at the hourglass while the example grid was occluded, and provides an indication whether overall delay durations were similar between conditions in which a delay was present.

Table 2: Outcomes of Bayesian Repeated-Measures ANOVAs, between the three delay conditions in Experiment 2 (constant, low variance, high variance).

	Outcome variable	BF_{10}
**A**	Example grid inspections	1.581
**B**	Fixations per inspection	0.278
**C**	Items placed per inspection	0.516
**D**	Completion time (s)	0.227
**E**	Errors per trial	0.163
**F**	Time spent waiting (s)	1.288

Table 3: Statistical outcomes for Bayesian paired samples t-tests between the three delay conditions in Experiment 2, corrected for three comparisons.

	Outcome variable	Condition	Comparison	BF_{10}
**A**	Example grid inspections	Constant	Low variance	1.673
		Constant	High variance	0.380
		Low variance	High variance	0.274
**B**	Fixations per inspection	Constant	Low variance	0.211
		Constant	High variance	0.172
		Low variance	High variance	0.233
**C**	Items placed per inspection	Constant	Low variance	0.465
		Constant	High variance	0.170
		Low variance	High variance	0.293
**D**	Completion time (s)	Constant	Low variance	0.174
		Constant	High variance	0.243
		Low variance	High variance	0.168
**E**	Errors per trial	Constant	Low variance	0.154
		Constant	High variance	0.154
		Low variance	High variance	0.154
**F**	Time spent waiting (s)	Constant	Low variance	1.022
		Constant	High variance	0.414
		Low variance	High variance	0.251

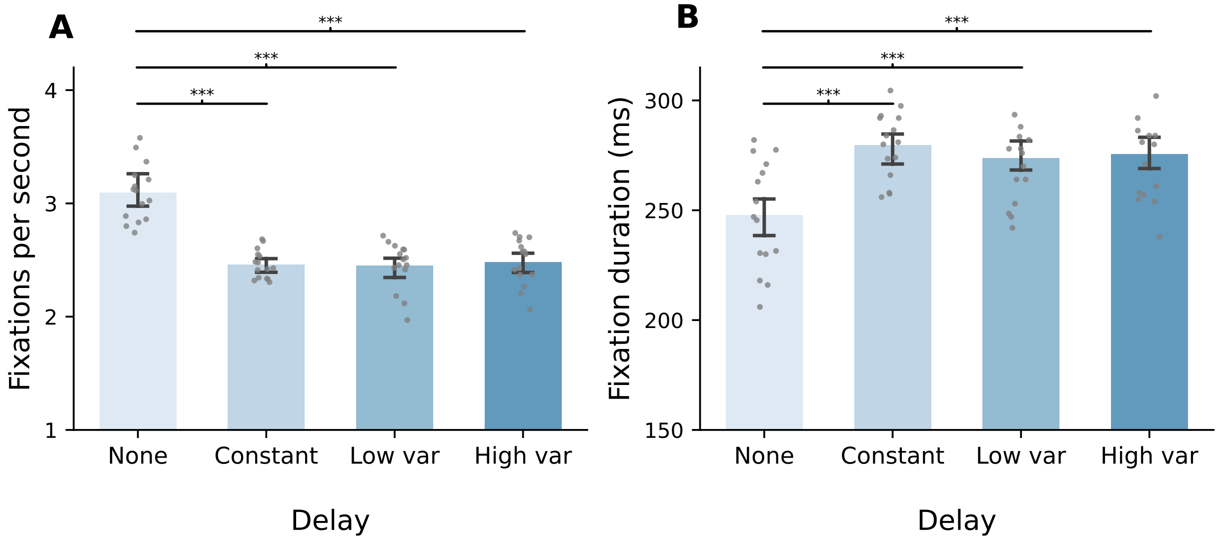

Figure 2: Barplots (mean ± 95% within-subjects CI) for each variable, per condition. Individual points represent within-participant aggregates. **A.** The number of fixations per second per trial. **B.** The median fixation duration in milliseconds per trial.
Note. Post-hoc paired samples t-tests (Bonferroni corrected); $^*p < .05$, $^{**}p < .01$, $^{***}p < .001$.

Table 4: Outcomes of Linear Mixed Effect model (LME) with completion time as dependent variable. Data from Experiment 2. Conditions were grouped into *no delay* (baseline condition) and *delay* (constant, low variance, high variance). LME models were run with the Lmer function from lme4 (Bates et al., 2015, version 1.1-35.1) using pymer4 (Jolly, 2018, version 0.8.1). Formula used:
`Completion time ∼ Inspections + Fixations + Errors + Condition + (Inspections + Fixations + Errors + Condition | ID).`

	β	2.5% CI	97.5% CI	SE	df	t	p	Sig.
(Intercept)	9.449	7.057	11.840	1.220	13.517	7.744	<.001	***
Example grid inspections	1.840	1.239	2.440	0.306	11.627	6.003	<.001	***
Fix. per insp.	0.492	0.326	0.658	0.085	12.539	5.803	<.001	***
Errors per trial	2.190	1.879	2.502	0.159	12.499	13.777	<.001	***
Condition (no delay - delay)	-4.151	-5.750	-2.552	0.816	13.785	-5.089	<.001	***

References

Bates, D., Mächler, M., Bolker, B., & Walker, S. (2015). Fitting Linear Mixed-Effects Models Using lme4. *Journal of Statistical Software*, *67*, 1–48. https://doi.org/10.18637/jss.v067.i01

Bays, P. M., Gorgoraptis, N., Wee, N., Marshall, L., & Husain, M. (2011). Temporal dynamics of encoding, storage, and reallocation of visual working memory. *Journal of Vision*, *11*(10), 6. https://doi.org/10.1167/11.10.6

Jolly, E. (2018). Pymer4: Connecting R and Python for Linear Mixed Modeling. *Journal of Open Source Software*, *3*(31), 862. https://doi.org/10.21105/joss.00862

Koevoet, D., Naber, M., Strauch, C., Somai, R. S., & Van der Stigchel, S. (2023). Differential aspects of attention predict the depth of visual working memory encoding: Evidence from pupillometry. *Journal of Vision*, *23*(6), 9. https://doi.org/10.1167/jov.23.6.9

Sahakian, A., Gayet, S., Paffen, C. L. E., & Van der Stigchel, S. (2023). Mountains of memory in a sea of uncertainty: Sampling the external world despite useful information in visual working memory. *Cognition*, *234*, 105381. https://doi.org/10.1016/j.cognition.2023.105381

van den Berg, R., Shin, H., Chou, W.-C., George, R., & Ma, W. J. (2012). Variability in encoding precision accounts for visual short-term memory limitations [Publisher: Proceedings of the National Academy of Sciences]. *Proceedings of the National Academy of Sciences*, *109*(22), 8780–8785. https://doi.org/10.1073/pnas.1117465109
